# Stress as a potential moderator of ovarian hormone influences on binge eating in women

**DOI:** 10.12688/f1000research.16895.1

**Published:** 2019-02-27

**Authors:** Natasha Fowler, Phuong T. Vo, Cheryl L. Sisk, Kelly L. Klump

**Affiliations:** 1 Department of Psychology, Michigan State University, 316 Physics Road, East Lansing, MI, 48824-1116, USA; 2 Neuroscience Program, Michigan State University, 293 Farm Lane, East Lansing, MI, 48824-1116, USA

**Keywords:** stress, ovarian hormones, binge eating, bulimia nervosa, binge eating disorder, estrogen, progesterone, women

## Abstract

Previous research has demonstrated significant associations between increased levels of ovarian hormones and increased rates of binge eating (BE) in women. However, whereas all women experience fluctuations in ovarian hormones across the menstrual cycle, not all women binge eat in response to these fluctuations, suggesting that other factors must contribute. Stress is one potential contributing factor. Specifically, it may be that hormone-BE associations are stronger in women who experience high levels of stress, particularly as stress has been shown to be a precipitant to BE episodes in women. To date, no studies have directly examined stress as a moderator of hormone-BE associations, but indirect data (that is, associations between BE and stress and between ovarian hormones and stress) could provide initial clues about moderating effects. Given the above, the purpose of this narrative review was to evaluate these indirect data and their promise for understanding the role of stress in hormone-BE associations. Studies examining associations between all three phenotypes (that is, ovarian hormones, stress, and BE) in animals and humans were reviewed to provide the most thorough and up-to-date review of the literature on the potential moderating effects of stress on ovarian hormone–BE associations. Overall, current evidence suggests that associations between hormones and BE may be stronger in women with high stress levels, possibly via altered hypothalamic–pituitary–adrenal axis response to stress and increased sensitivity to and altered effects of ovarian hormones during stress. Additional studies are necessary to directly examine stress as a moderator of ovarian hormone–BE associations and identify the mechanisms underlying these effects.

## Introduction

Binge eating (BE) is a core symptom of many eating disorders, such as anorexia nervosa (AN), bulimia nervosa (BN), and binge eating disorder (BED) ^1^. BE is defined as repeated overconsumption of an objectively large amount of food (for example, >1000 calories) with a loss of control over the eating ^2^.

Although the etiology of BE remains largely unknown, emerging data suggest a role for ovarian hormones ^3– 7^. Indeed, both animal ^8– 11^ and human ^3– 7^ studies suggest that changing levels of both estrogen and progesterone likely contribute to risk for BE and possibly its predominance in females. Interestingly, whereas all women experience fluctuations in ovarian hormones, not all women develop BE. The prevalence of BE in the population ranges from 1.5% ^12^ to 26% ^13^, suggesting that there are likely moderators of the hormone-BE association that explain why some women binge eat in response to hormone fluctuations whereas others do not.

This review aimed to explore one such moderator—stress—defined in this review as the psychological perception of a stressful life event or the physiological response to a stressful life event, which is measured via hypothalamic–pituitary–adrenal (HPA) axis responsivity—and its impact on hormone-BE associations in women. Stress is an ideal candidate as a moderator of these associations. BE increases with increasing levels of stress ^14– 17^, and stress responsivity varies across the menstrual cycle in women ^18^. Thus, ovarian hormone–BE associations may be particularly strong in women with higher stress levels. If this is the case, then stress may account for why some, but not all, women binge eat in response to hormonal risk.

This review briefly describes the literature linking ovarian hormones to BE in women and reviews the potential role of stress responses in ovarian hormone–BE associations. Whenever possible, data from animal and human studies are explored to provide the most comprehensive picture of stress effects. Owing to the effects of starvation on ovarian hormones (for example, lack of cyclicity and loss of monthly cycles), most studies have not examined ovarian hormone effects on AN. For the purpose of this review, studies were limited to human and animal models of BE, BED, and BN. We end by suggesting areas for future research with the overarching goal of informing etiological models to understand why some, but not all, women binge eat during high-risk hormonal periods.

## Ovarian hormones and binge eating

### Animal studies

Animal studies provide some of the strongest support for ovarian hormone influences on eating behavior. Findings dating back more than 40 years show that estrogen has anorexic effects on food intake, with higher levels decreasing food intake and lower levels increasing food consumption ^8^. These data come from strong experimental studies in which the ovaries are removed—via ovariectomy (OVX)—and then hormones are re-administered exogenously. Following OVX, rats, mice, and monkeys ^19– 23^ significantly increase chow (that is, nutritive food) intake and body weight, which normalize following exogenous estradiol treatment ^8, 11, 24^. Treatment with progesterone alone does not directly affect food intake ^25– 28^, but progesterone antagonizes the effects of estradiol and increases food intake in OVX rats ^11, 24^. Fluctuations in food intake across the estrous cycle are also present in rodents, such that food consumption increases in non-ovulatory phases (for example, diestrous) and decreases during ovulation (that is, estrous) ^8, 29– 34^. Together, these data suggest that estrogen exerts direct, inhibitory effects on food intake while progesterone has stimulatory effects in the presence of estrogen.

Unfortunately, comparatively fewer animal studies have examined ovarian hormone effects on BE per se. Results thus far replicate those for chow intake ^9– 11, 24^. BE is typically assessed in these studies by exposing animals to intermittent (for example, 3 days per week) access to palatable food (PF) (that is, high-fat/-sugar food). Using these models, studies have shown that rats undergoing OVX in adulthood significantly increase binge-like eating ^9, 11^ that is reversed by estradiol treatment ^11, 35^ (and combination estradiol/progesterone treatment in some studies ^24^). Binge-like eating also increases in intact animals across the estrous cycle when estradiol levels are low (that is, diestrous) and decreases when estradiol levels are high (that is, estrous) ^10^.

### Human studies

Human research generally corroborates findings from animal studies. In women, it is difficult to directly manipulate hormone levels, but changes in BE across the menstrual cycle have been examined as a quasi-experimental design for understanding hormone effects. In these studies, changes in food intake and BE that are predicted by natural (and monthly) changes in hormones are used as an indication of longitudinal and predictive associations between ovarian hormones and BE. As in animal findings, food intake in women decreases with increasing estradiol levels and low progesterone levels (that is, follicular phase) ^36– 38^ and increases when both hormone levels are high (that is, mid-luteal phase) ^36– 38^. Nearly identical patterns of effects have been observed for BE ^4– 7^. When estradiol levels are high and progesterone levels are low (for example, follicular phase and ovulation), rates of BE and emotional eating (EE) (that is, a significant correlate and predictor of BE, defined as overeating in response to negative emotions) are low ^3– 5, 39^. Conversely, when both estradiol and progesterone levels are high (for example, mid-luteal phase), rates of BE/EE significantly increase ^3– 5, 39^. Natural fluctuations in ovarian hormones across the menstrual cycle seem to account for these effects, as higher levels of both estradiol and progesterone predict increased levels of BE and EE across the cycle ^4, 39, 40^. These associations between hormones and BE/EE are present in community ^5, 39– 41^ and clinical ^4, 6^ samples of women, although effects appear stronger in clinical samples ^4, 6^.

Overall, animal and human studies have significant predictive (and, in animal studies, causal) associations between ovarian hormones and changes in food intake and BE in females ^3– 11^. Although the factors contributing to these associations remain unknown, it seems likely that changes in gene transcription are major contributors. Ovarian hormones are steroid hormones whose primary function is to regulate gene transcription/expression (and, thereby, protein synthesis) in the neurobiological systems (for example, serotonin and dopamine) known to be disrupted in eating disorders ^42, 43^. Thus, it is possible that during risky hormonal milieus (for example, non-estrous or mid-luteal phases) there is differential regulation of risky or protective genes for BE in key neurobiological systems ^44^. Data from twin studies indirectly support this possibility, as the heritability of eating disorder symptoms varies dramatically across the menstrual cycle ^40^ and other hormonal “events” (for example, puberty) ^45– 47^, and suggest that ovarian hormone activation and de-activation are associated with changes in genetic risk. This is likely one set of moderators that contribute to hormone-BE associations, whereby associations between hormones and behavioral phenotypes vary by the presence/absence of risk/protective genes. Nonetheless, other moderators likely also contribute and may also interact with ovarian hormones to influence behavior. The psychological and physiological responses to stress are strong candidates in this regard.

## Stress and binge eating

### Animal studies

Stress is a common precipitant to BE ^14– 17^. Animal models repeatedly demonstrate that rats, mice, and non-human primates subjected to stress exhibit hyperphagia of general food intake ^48– 51^ and binge-like eating ^48, 52– 54^. Results are consistent across various forms of stress tasks (for example, shock stress, restriction/refeeding, and chronic social defeat) ^50^. For example, following repeated bouts of foot shock, female BE-prone (BEP) rats consumed significantly larger amounts of sucrose than BE-resistant (BER) rats ^53^. BEP rats also consumed sucrose more rapidly and demonstrated compulsive-like intake of sucrose following stress compared with BER rats ^53^. Mice exhibit similar increases in food intake following chronic subordination stress (that is, repeatedly exposing a submissive animal to an aggressive, dominant animal) that becomes exacerbated in response to food restriction (a known precipitant to overeating) ^48, 52, 55^ or acute social defeat ^49^. Restriction/refeeding stress (that is, access to PF diet followed by food deprivation and then refeeding of PF diet) also induces a hyperphagic rebound upon refeeding of PF ^56^.

Stress effects on eating behavior appear dependent upon BE phenotype. Prior to stress exposure and intermittent PF access, both BEP and BER animals exhibit increases in plasma corticosterone levels ^53^. However, following repeated exposure to stress, BEP animals begin to display a blunted corticosterone response to stress ^53, 57^, indicative of a hypoactive HPA-axis response, compared with BER animals that display the typical post-stress increase in plasma corticosterone ^58^. Because differences in stress response between the phenotypes do not appear until stress and PF access induction, it is possible that there are inherent physiological and neurobiological differences between the two BE phenotypes that become exacerbated upon exposure to stress.

One potential neurobiological difference that may contribute to the development of the two phenotypes is the corticotropin-releasing hormone (CRH) system within hypo- and extra-thalamic brain regions. Chronic stress increases mRNA expression of CRH and CRH receptors in brain regions associated with food intake (for example, paraventricular nucleus) and stress response (for example, central amygdala) ^57, 58^. However, increases in CRH levels are significantly higher in BEP compared with BER rats following stress ^57^. CRH triggers release of adrenocorticotrophic hormone from the pituitary, which stimulates release of glucocorticoids (for example, corticosterone) from the adrenal glands ^18^. Therefore, elevated CRH levels increase corticosterone levels in animals. Increased corticosterone levels are associated with increased anxiety-like behaviors ^59, 60^ and concordant increases in food intake ^58, 61^. These results suggest increased susceptibility to stress in BEP versus BER animals that may alter underlying neural circuitry involved in stress and food intake regulation in order to perpetuate the BE phenotype.

### Human studies

In humans, the majority of studies examining acute stress effects on BE in the laboratory use physical (for example, cold pressor test) ^62^, psychosocial (for example, Trier Social Stress Test [TSST]) ^63^, or cognitive (for example, Stroop test) ^64^ stress tasks. A smaller number of studies also examine daily experiences of stress by using self-report questionnaires (for example, Perceived Stress Scale) ^65^ or biological measures of stress responses (for example, salivary or urinary cortisol). Finally, chronic stress has been examined by studying samples with known or self-reported exposure to chronic stress (for example, trauma and abuse) measured via self-report questionnaires (for example, Childhood Trauma Questionnaire) ^66^ or longitudinal biomarkers of stress (for example, daily salivary cortisol assays).

Consistent with the effects found in animal studies, effects of acute stress on eating behavior exist in women ^67– 69^ and appear dependent upon BE status. Following acute psychosocial (that is, TSST) or physical (that is, cold pressor task) stress, women who binge eat rated foods as more palatable ^67^, displayed increased motivation toward food ^68, 70, 71^, and showed an increased desire to binge eat ^71^ compared with non-stressed BED and control women. Food intake ^72– 74^ and BE ^75, 76^ also increase following exposure to acute stress in women who binge eat compared with control women. Elevated basal cortisol levels ^69, 77– 80^ and a blunted acute stress response ^81, 82^, indicative of chronic stress exposure and altered HPA-axis responses, are also observed in women who binge eat ^18^. Interestingly, women who binge eat are more likely to have experienced chronic life stress (for example, abuse and trauma) ^77– 80, 83, 84^ and often exhibit a hypoactive HPA-axis response ^17^ compared with non-BE women, indicating potentially important associations between chronic stress or altered stress responses (or both) and BE in women.

However, not all women binge eat in response to stress. Currently, it is unknown why stress increases the risk for BE in some, but not all, women. Studies in humans have yet to test the effects of CRH mRNA and receptor expression observed in animals (described above). However, studies suggest that differential neural activation in women who binge eat may partially explain this discrepancy. Following the presentation of food cues from pre- to post-stress induction, women with BN exhibited decreased activation in brain regions associated with emotion regulation (for example, anterior cingulate cortex, amygdala, and ventromedial prefrontal cortex) ^85, 86^ and self-focus (for example, precuneus) ^68^ compared with healthy controls. Among the women who binge eat, those with greater de-activation in these brain regions reported increased stress levels prior to BE ^85, 86^. This differential response to stress on eating behavior suggests that, as in animal studies, inherent differences in neural circuitry following stress may increase susceptibility to BE in a subset of women. These findings highlight the need for additional studies that elaborate upon the mechanisms through which stress might influence BE.

## Stress and ovarian hormones

### Animal studies

Ovarian hormones play a critical role in modulating the stress response in animals and humans. Specifically, ovarian hormones regulate HPA-axis functioning via antagonistic effects on glucocorticoids ^87^. At high levels of estradiol, as in ovarian hormone regulation of eating behavior, glucocorticoid receptor levels are decreased ^88^, HPA-axis responsivity is enhanced ^18^, and animals exhibit fewer stress-like (for example, anxiety) behaviors ^88, 89^. Conversely, when either progesterone alone or both ovarian hormones are elevated, glucocorticoid levels increase ^90– 92^, HPA-axis activity decreases ^18^, and animals exhibit more stress-like behaviors ^88^.

It should be noted that when estradiol is administered to OVX animals without progesterone, some studies have reported the expected decreases in glucocorticoid levels ^90, 93, 94^ whereas others have reported increases in glucocorticoid levels ^91, 95^. Differing results may be due to different responses based on estradiol dose, such that the administration of high physiological levels of estradiol produces protective effects against stress ^94^, whereas the administration of lower physiological levels may not produce protective effects ^91, 96^. Additional research is needed to confirm these impressions and identify the potential mechanisms underlying these dose-response relationships. However, initial results ^18, 87– 89, 97– 99^ are promising in supporting a protective role for estrogen against stress and an antagonistic role for progesterone ^96^ against estrogen, similar to the effects observed with eating behavior. Notably, during more severe and chronic levels of stress, ovarian hormone functioning can shut down because of antagonistic effects of elevated glucocorticoid (for example, cortisol) levels on ovarian hormones ^100^. In these cases, the estrous/menstrual cycle becomes irregular and/or cycling stops. Because the hypothalamic–pituitary–gonadal axis is completely shut down, these effects cannot account for the hormone-BE associations that are observed in regularly cycling animals and humans and that are the focus of this review. Nonetheless, these extreme stress–hormone interactions would be important to examine in populations of women with BE who are no longer menstruating and who have experienced high levels of chronic stress.

Interestingly, ovarian hormone and glucocorticoid (for example, cortisol) receptors colocalize in brain networks ^101^ and binding sites of promotor regions of similar genes responsible for regulating stress and food intake ^87, 102, 103^. This colocalization creates competition for binding sites and leads to increased susceptibility for stress–ovarian hormone interactions in females across ovarian hormone levels, such that at high estradiol and low progesterone levels, glucocorticoid levels are low compared with when both ovarian hormones are elevated ^104^.

### Human studies

Findings from human studies corroborate data from animal studies suggesting associations between ovarian hormones and stress responses in women. In women, ovarian hormones modulate HPA-axis functioning via antagonistic effects on glucocorticoids ^18^. For example, women with ^105^ and without ^104, 106– 108^ BE perceive stimuli (for example, giving a speech) as less stressful when estradiol levels are high and progesterone levels are low, compared with when both hormone levels are elevated. Results from community samples of women also suggest that basal plasma cortisol levels and adrenal production of cortisol post-stress are higher post-ovulation versus pre-ovulation ^104^.

Ovarian hormones also regulate neural activity in key subcortical regions of stress circuitry (for example, amygdala, hippocampus, and hypothalamus) ^109– 111^ such that when both ovarian hormones are elevated (for example, during the mid-luteal phase), activation of stress circuitry increases compared with when estradiol is elevated and progesterone levels are low (for example, during the follicular phase) ^112, 113^. Studies examining the effects of progesterone on stress are limited, but current theories suggest an antagonistic role for progesterone against the protective benefits of estrogen, particularly at peak progesterone levels ^92, 114, 115^. Taken together, these findings suggest a protective role for estrogen against stress and a possible antagonistic role for progesterone toward estrogen.

## Stress as a moderator of ovarian hormone–binge eating associations

As previously stated, not all women binge eat in response to ovarian hormones or stress. Given the associations between ovarian hormones, stress, and BE (described above), it is possible that stress moderates ovarian hormone–BE associations, strengthening these associations in highly stressed women. In these women, risky hormonal milieus (that is, high levels of estradiol and progesterone) may enhance psychological (for example, perceived levels of stress) and biological (for example, elevated glucocorticoid levels) stress responses and lead to even higher levels of BE compared with women with lower stress levels.

Unfortunately, to date, no studies have examined whether stress moderates hormone-BE associations in humans. Studies have examined this association in animals and found significant moderating effects of stress on ovarian hormone–BE associations ^10, 116– 118^. Acute stress (that is, frustrative non-reward) significantly increased PF consumption (by 30% to 40%) in BEP rats studied during low estradiol phases (for example, diestrous) as compared with non-stressed rats and stressed rats studied during high estradiol phases (for example, estrous) ^10^. Stress (for example, food-restriction stress) also significantly increases PF consumption in OVX rats compared with non-stressed OVX rats ^11^. Treatment with exogenous estrogen and progesterone attenuates these stress-induced increases in PF consumption in OVX animals ^11^, although the magnitude of attenuation seems dependent on the intensity of the stressor ^11, 116– 118^. Together, these results suggest that ovarian hormones exert protective effects against increased BE at low stress (for example, restriction) conditions. However, under high stress/chronic stress conditions, the protective effects of estrogen may be decreased or even reversed.

## Conclusions

The etiology of BE remains to be fully elucidated; however, previous studies have repeatedly established ovarian hormones ^4, 6, 8^ and stress ^14– 17^ as critical contributing factors to BE in animals and women. Studies also demonstrate substantial regulation of the stress response by ovarian hormones with patterns mimicking those found for ovarian hormone influences on BE (that is, more significant stress effects at elevated versus low ovarian hormone levels) ^101– 104, 119^. Stress may also initiate different physiological and neural responses in animals ^53, 57^ and women ^68, 85, 86^ who do and do not binge eat, which reinforce the BE phenotype. These differences may become exacerbated at varying ovarian hormone levels. Lastly, stress increases PF consumption in female rats with lower estradiol levels or who have undergone OVX. Overall, the current literature provides strong support for individual associations among stress, ovarian hormones, and BE in both women and animals, and emerging data from animal studies indirectly support stress as a moderator of ovarian hormone influence over general food intake.

Clearly, more evidence is needed to more firmly establish stress as a moderator of hormone-BE associations. For animal studies, a key starting place would be to examine stress and hormone effects on binge-like eating rather than general food intake. OVX animal models should also be used as strong experimental controls to examine binge-like eating and PF consumption before and after both stress exposure and exogenous hormone administration. Lastly, studies should examine effects of multiple stressor types (for example, foot shock and restraint stress) to determine the moderating effect of different stressor types on ovarian hormone–BE associations.

Human studies need similarly strong quasi-experimental designs. First, studies should examine daily stress, hormone, and BE levels to examine stress by hormone interactions on BE. The menstrual cycle is one model in which to test these effects due to natural fluctuations in ovarian hormones. Oral contraceptive (OC) use can also be used to examine stress by hormone interactions on BE. Because OCs consist of active and inactive pill phases, human studies can mimic hormonal manipulation paradigms seen in animal literature (for example, OVX and hormone administration). In addition to self-reported stress, lab-based stressors (for example, TSST) should be used to assess subjective and objective responses to stress as they relate to hormone-BE associations.

Although this review focused on women of reproductive age, data suggest that other developmental periods (for example, puberty ^45– 47^ and menopause ^120– 123^) may be important for BE risk. Puberty and menopause are critical developmental periods during which dramatic increases or decreases in ovarian hormone levels can activate or de-activate the protective effects of estrogen on stress responsivity ^124– 128^ and BE ^45– 47, 120– 122^. Future studies are needed to better understand the role of stress and ovarian hormone interactions on BE risk during these risky developmental hormonal milieus.

Finally, relatively little is known about the neurobiological mechanisms underlying the independent or interactive effects of stress and ovarian hormones on BE. Initial studies suggest that increased expression of CRH in brain regions associated with food intake and stress response ^57, 58^ and decreased activation in limbic and frontal regions following stress ^68, 85, 86^ may be important. However, more studies using neuroimaging (for example, functional magnetic resonance imaging) in humans and brain processing (for example, optogenetics) in animals are needed to explore these possibilities.
